# Numerical Response of Migratory Shorebirds to Prey Distribution in a Large Temperate Arid Wetland, China

**DOI:** 10.1155/2016/1297603

**Published:** 2016-12-13

**Authors:** Yamian Zhang, Yi Zhu, Aojie Zuo, Li Wen, Guangchun Lei

**Affiliations:** ^1^School of Nature Conservation, Beijing Forestry University, Beijing 100083, China; ^2^Science Division, Office of Environment and Heritage, Sydney, NSW 2000, Australia

## Abstract

Wuliangsuhai Lake provides important breeding and stopover habitats for shorebirds. The health of this wetland ecosystem is rapidly deteriorating due to eutrophication and water pollution and environmental management is urgently needed. To explore the connections among ecosystem health, prey density, and shorebird populations, we conducted surveys of both the benthic macroinvertebrates and shorebirds in the shorebird habitat of the wetland during the 2011 autumn migration season. The abundance of both shorebirds and benthic macroinvertebrates varied significantly in both space and time. Our data showed a clear association between shorebird populations and the density of benthic macroinvertebrates, which explained 53.63% of the variation in shorebird abundance. The prey density was strongly affected by environmental factors, including water and sediment quality. Chironomidae were mainly found at sites with higher total phosphorus, but with lower sediment concentrations of Cu. Lymnaeidae were mainly found at sites with a higher pH, lower salinity, and lower concentrations of total phosphorus and Cu. Habitats with very high concentrations of total phosphorus, heavy metals, or salinity were not suitable for benthic macroinvertebrates. Our findings suggest that the reductions of nutrient and heavy metal loadings are crucial in maintaining the ecological function of Wuliangsuhai as a stopover habitat for migratory shorebirds.

## 1. Introduction

Shorebirds are known to forage extensively on benthic macroinvertebrates [1, 2] and the abundance of benthic macroinvertebrates is likely to be crucial for the short- and long-term survival of shorebirds given their high energy demands during migration and the breeding season [3–5]. Shorebirds are therefore predicted to show numerical and functional responses to changes in the abundance of their prey [6, 7]. Migratory shorebirds depend on stopover sites along their migration routes to rest and replenish their energy reserves [8, 9]. Many factors affect the distribution of their prey species, including water and sediment quality and aquatic plants [10, 11], which, in turn, affects the aggregation patterns of foraging shorebirds.

Wuliangsuhai Lake is a key wetland in the vast, arid region of northwest China and provides important breeding and staging habitats for shorebirds in the East Asian–Australasian Flyway [12]. However, as the major area for water storage and the discharge of agricultural drainage in the Yellow River Bend Region [13], the wetland is highly eutrophic [14]. Subsequently, the structure and function of this wetland have gradually changed—for example, the rapid expansion of* Phragmites *into open water areas has been well documented [15, 16]. Structural changes in the flora may modify the distribution and abundance of benthic macroinvertebrates [17] and ultimately affect the wetland's function as a feeding ground for shorebirds. However, the relationship between shorebirds and their prey at Wuliangsuhai Lake has not been investigated on any scale and the numerical response of shorebirds to the variation in benthic macroinvertebrates remains unknown.

This study investigated the spatial association of shorebirds and their food resources within Wuliangsuhai Lake. We conducted regular shorebird surveys at three main foraging sites along environmental gradients during the 2011 autumn migration season and sampled the benthic macroinvertebrates and collected data on environmental variables along the bird survey transects. We aimed to answer two specific questions: (1) what are the important environmental factors for benthic macroinvertebrates and (2) how do shorebirds respond to the changes in their food sources (i.e., the benthic macroinvertebrate density)? This information is crucial in the management of the wetland, particularly as a habitat for migratory waterbirds.

## 2. Materials and Methods

### 2.1. Study Site

**Figure 1:**
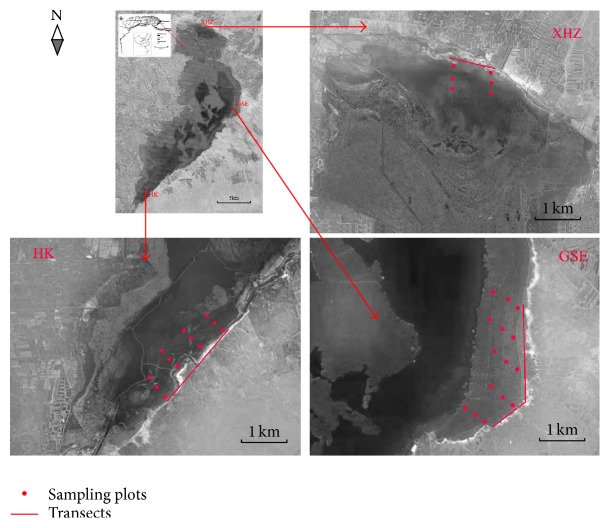
Location of Wuliangsuhai Lake and the arrangement of shorebird survey transects (lines) and sampling plots for macroinvertebrates and environmental variables (dots) within the study areas of Gesuer (GSE), Hekou (HK), and Xiaohaizi (XHZ).

Wuliangsuhai Lake is located in Inner Mongolia, China (40°46′–41°05′N, 108°42′–108°58′E) (Figure 1), and has an area of 293 km^2^ and a mean water depth of about 1 m [17]. The prevailing climate is continental with a low mean annual rainfall (220 mm), high annual evaporation (1,502 mm), and a mean annual temperature of 7.0°C [18]. The wetland is frozen from November to March and the frost-free period averages 152 days [19]. The main water source is agricultural drainage from the Hetao Irrigation Area. Recent studies have shown that the concentrations of total nitrogen (TN) and total phosphorus (TP) range from 1.44 to 19.31 and 0.024 to 0.057 mg/L, respectively, indicating that the wetland is highly eutrophic [20, 21].

About 50.9% of the wetland surface area is covered by emergent plants and 43.7% by submerged plants; the remaining 5.4% consists of sandbars and shoals [22]. The wetland is recognized as one of the most important areas for birds in the vast, arid region of northwest China. A total of 241 bird species has been recorded from 17 orders and 49 families, including black stork* (Ciconia nigra)*, relict gull* (Larus relictus)*, and mute swan* (Cygnus olor)* [23]. Shorebirds of global conservation importance include far eastern curlew* (Numenius madagascariensis)*, Asian dowitcher* (Limnodromus semipalmatus)*, black-tailed godwit* (Limosa limosa)*, and Eurasian curlew* (Numenius arquata)* [12].

### 2.2. Bird Survey

To investigate the possible associations between shorebirds and benthic macroinvertebrates and to test whether there were variations in the abundance of shorebirds in space and time, data on shorebirds were collected from biweekly surveys during the 2011 autumn migration (late August to early November 2011; eight observation periods) [12]. For the shorebirds, we focused on the families Scolopacidae and Charadriidae because these are the main groups of shorebirds that visit Wuliangsuhai Lake annually [12, 24]. The surveys were conducted at three main shorebird foraging sites in the wetland: Xiaohaizi (XHZ), Gesuer (GSE), and Hekou (HK) (Figure 1). The sites were chosen to include a range of environmental gradients (e.g., water and sediment quality), although the choice of sites was also influenced by accessibility and safety considerations. XHZ is located in the northernmost part of the wetland, near where drainage water flows into the lake. This area is largely covered by emergent plants, with small patches of shallow water and sandbars. GSE is located on the eastern shore and is mainly covered by emergent and submerged plants; it has the largest proportion of shallow water (depth < 1 m) among the three sampling sites. HK is located in the southernmost part of the wetland where the water flows into the Yellow River. It consists of a large area of open water with an area of shallow water near the shore. All three sampling sites provide foraging and resting habitats for shorebirds [12, 22]. A fixed transect with variable lengths was established at each sampling site. Based on the size of the habitat (about 5 km^2^ at GSE, 4 km^2^ at HK, and 2 km^2^ at XHZ), the transect length was 4, 3, and 1 km for GSE, HK, and XHZ, respectively. Each survey was conducted by walking the length of the transect parallel to the lakeshore at a constant speed; the perpendicular searching distance was 0.6 km. Using binoculars (8 × 42) and telescopes (Swarovski ATS 80 HD 20−60 × 80), we counted and recorded all the shorebirds (in sight or heard). To increase detectability, the surveys were carried out in daytime on clear days and there were at least two fully trained observers for each transect. The variability in observer error was minimized by using the same observers whenever possible throughout the study period. To avoid repeated counting, the three transects were surveyed simultaneously. Visual and/or verbal communication enabled us to avoid duplicate recordings of the same flock of shorebirds by at least two observers. Shorebirds that flew forward were excluded and all the shorebirds present were identified to species level.

### 2.3. Benthic Macroinvertebrate Survey

Benthic macroinvertebrate samples were collected in the shallow water area of the lake (along the shorebird survey transects) using PVC pipes (7.14 cm diameter) on 17–19 September and 16-17 October 2011. The sampling dates were chosen to reflect the shorebird phenology and represent the “peak” and “postpeak” dates in the autumn migration. As most shorebird species at Wuliangsuhai Lake are unable to forage on prey that is distributed in sediment deeper than 10 cm [24], only the top sediment layer was collected and analyzed. This depth was sufficient to capture most if not all benthic macroinvertebrates that serve as shorebird prey at Wuliangsuhai Lake. Sediment cores were taken parallel to the transects at regular 100 m intervals and sampling was repeated every 1 km (Figure 1). The number of sediment cores collected during each sampling period for the three sampling sites was six, 15, and 12 for XHZ, GSE, and HK, respectively, giving a total of 66 samples. Duplicate samples were collected and the sediment cores were sliced into top (0–5 cm) and bottom (5–10 cm) layers, washed, and sorted using a 63 *µ*m sieve [25]. The remaining material was preserved in 95% ethanol and examined microscopically. All individual organisms were counted and because the counts of benthic macroinvertebrate species other than Chironomidae and Lymnaeidae (e.g., the larvae of Tabanidae) were very low, they were grouped together and labeled as “Others” to give three major groups of benthic macroinvertebrate: Chironomidae, Lymnaeidae, and Others. We followed the methods of Epler to identify chironomid larvae [26]. The methods of Merritt et al. [27] and Liu et al. [28] were used to identify other species.

### 2.4. Substrate Quality and Water Quality

We collected substrate samples at each benthic sampling location using PVC pipes with the same diameter as the macroinvertebrate sampling tubes. The substrate sampling was conducted from 17 to 19 September 2011 and a total of 66 substrate samples were collected, the same number of samples as for the benthic macroinvertebrates. The substrate cores were also divided into top (0–5 cm) and bottom (5–10 cm) layers. The samples were analyzed in the laboratory of the Institute of Geographic Sciences and Natural Resources Research, Chinese Academy of Sciences (Beijing, China) for nitrogen (N), phosphorus (P), organic matter (OM), and heavy metals (As, Co, Cu, Li, Ni, and Hg). The N was determined using an elemental analyzer (Vario MACRO cube, Elementar, Germany). The OM was determined by titration with K_2_Cr_2_O_7_. The concentrations of P, Co, Cu, Li, and Ni were determined by inductively coupled plasma optical emission spectrometry using an Optima 5300 DV spectrometer (Perkin-Elmer, USA) and the concentrations of As and Hg were determined by inductively coupled plasma mass spectrometry (Elan DRC-e, USA). The substrate was classified into three types based on the particle diameter: sand, sand-mud, and mud.

Water quality measurements were taken at the same time as the benthic macroinvertebrate samples from 17 to 19 September 2011. The in situ water temperature, pH, and dissolved oxygen were measured with a YSI Professional Plus handheld multiple parameter meter (YSI, USA). Chlorophyll a (Chl a) was measured on-site with a Hydrolab MS 5 instrument (HACH, USA). Water samples were collected and preprocessed on site for other parameters, including salinity, TN, and TP. All water samples were processed in the laboratory of Urat Front Banner Environmental Protection Monitoring Station (Inner Mongolia, China) on the sampling day using a TU-1810 UV-VIS spectrophotometer (Persee Incorporated, China).

### 2.5. Statistical Analyses

We used the nonparametric Kruskal-Wallis test to compare the differences in the abundance of shorebirds, benthic macroinvertebrates, and environmental variables between sampling sites. The Mann–Whitney* U* test was applied to the benthic macroinvertebrates and quality of substrates collected from different layers to compare the distribution of the variables at different depths.

We applied multiple permutation linear regression to explore the relationship between the abundance of shorebirds and benthic macroinvertebrates. Model selection was based on the resultant* p* value and the Akaike information criterion [29]. A one-sample Kolmogorov-Smirnov test was used to test the normality of shorebird abundance. The test indicated that the data slightly violated normality (*p* = 0.087). In consideration of the small sample size and possible outliers, we used the permutation test instead of the normal theory test for statistical inference [30].

The distribution variation of benthic macroinvertebrates with environmental gradients was examined using canonical correspondence analysis (CCA) [31] on the log⁡(*x* + 1)-transformed benthic macroinvertebrate density because this allows a quick appraisal of how the community composition varies with environmental gradients. The densities of the three groups of benthic macroinvertebrates at GSE, HK, and XHZ in relation to environmental data were included in the analysis. We pooled the benthic macroinvertebrate density data of the three sediment cores in one sampling transect and used it as the density of the site in the CCA analysis. Thus, we had a total of five sites at GSE, two sites at XHZ, and four sites at HK. Before the CCA, we tested the correlations among these variables using Pearson's correlation coefficient and the strongly correlated variables (i.e., Pearson's *r* > 0.75) were excluded, including dissolved oxygen, Chl a, TN, OM, N, As, Co, Li, Ni, and Hg. A permutation test was used to investigate the correlations between benthic macroinvertebrates and environmental variables. All statistical analyses were performed in R3.2.2 [32].

## 3. Results

### 3.1. Spatial and Temporal Distribution of Shorebirds

**Figure 2:**
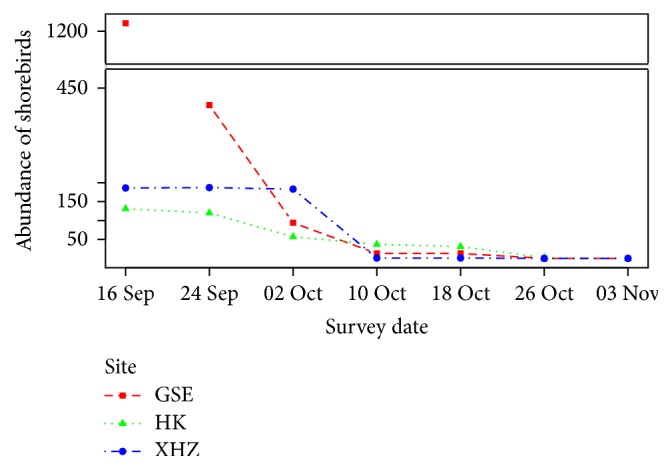
Number of shorebirds observed at the three sampling sites of Gesuer (GSE), Hekou (HK), and Xiaohaizi (XHZ) during the autumn migration.

A total of 27 shorebird species with over 3,300 individuals were recorded in the 2011 autumn migration season at Wuliangsuhai Lake. The three most abundant species, black-tailed godwit, spotted redshank* (Tringa erythropus)*, and Pacific golden plover* (Pluvialis fulva)* comprised 83% of the shorebird population; Northern lapwing* (Vanellus vanellus)*, Kentish plover* (Charadrius alexandrinus)*, and black-winged stilt* (Himantopus himantopus)* made up another 13% of the shorebirds. The total number of shorebirds decreased sharply after September and then decreased gradually until the end of migration season on 6 November 2011 (Figure 2).

The number of shorebirds at the three sampling sites was significantly different (Kruskal-Wallis test, *p* = 0.030). At the early stage of the study (in September), the highest number of shorebirds was observed at GSE, followed by XHZ and HK, with 68.57, 16.65, and 14.78% of the total count, respectively. More birds were counted at GSE and XHZ than at HK before 10 October 2011, after which the opposite distribution pattern was observed (Figure 2).

### 3.2. Spatial and Temporal Distribution of Benthic Macroinvertebrates

The most abundant prey in the wetland during both the peak and postpeak shorebird migration was Chironomidae (Table 1).

**Figure 3:**
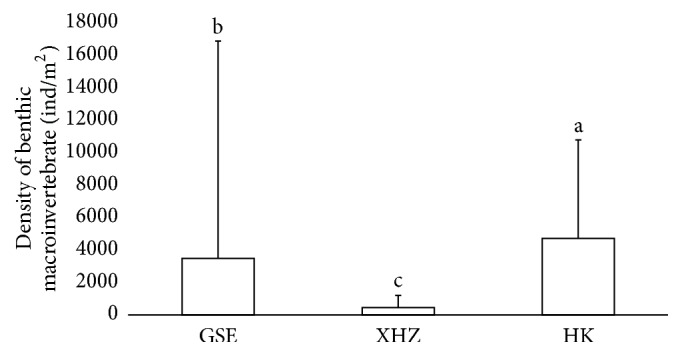
Density of benthic macroinvertebrate (mean ± SD) at the three sampling sites of Gesuer (GSE), Xiaohaizi (XHZ), and Hekou (HK) (*n* = 30, 12, and 24 for GSE, XHZ, and HK, respectively).

**Figure 4:**
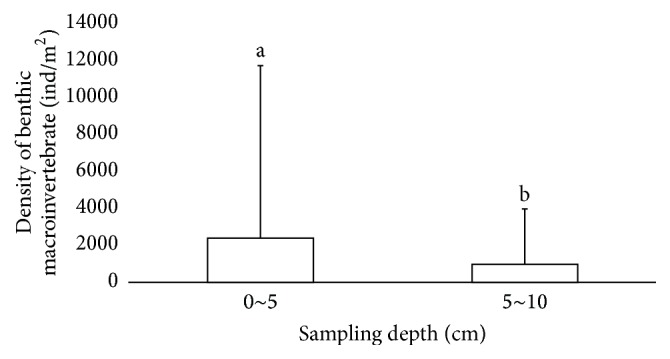
Density of benthic macroinvertebrates (mean ± SD) at sediment depths of 0–5 and 5–10 cm (*n* = 66).

**Figure 5:**
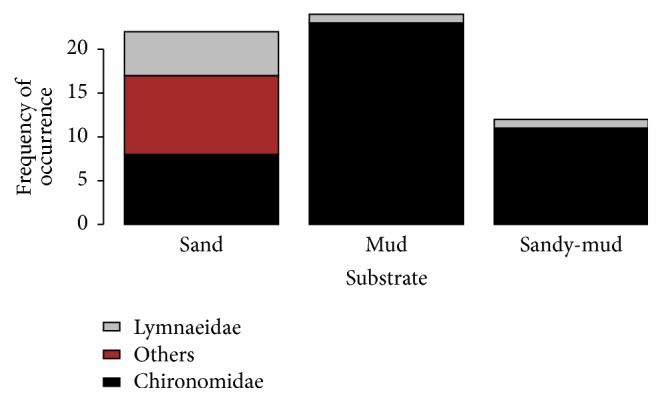
Frequency of occurrence of benthic macroinvertebrates in different substrate types during benthic coring within each sample plots (*n* = 66).

There were large spatial (horizontal and vertical) and temporal variations in the distributions of benthic macroinvertebrates. Spatially, the Kruskal-Wallis test showed that the density of benthic macroinvertebrates varied significantly between the three sampling sites (*p* < 0.001), with HK having the highest density and XHZ the lowest (Figure 3). The Mann–Whitney* U* test showed a significant vertical difference, with a significantly higher density of benthic macroinvertebrates in the top layer of sediment than in the bottom layer (*p* < 0.001) (Figure 4). Temporally, the average density of benthic macroinvertebrates in mid-September 2011 (5460 indiv./m^2^) was substantially higher than in mid-October 2011 (1000 indiv./m^2^) (*p* = 0.005). Although Lymnaeidae were largely found in coarse sediments (sand), Chironomidae were mainly found in fine sediments (mud). Sandy-mud could still support a certain amount of Chironomidae (Figure 5).

### 3.3. Response of Shorebirds to the Distribution of Benthic Macroinvertebrates

Permutation linear regression analyses showed that among the three groups of benthic macroinvertebrates, only Chironomidae showed a positive correlation with shorebird abundance (*p* = 0.049). The model selection procedure using the Akaike information criterion indicated that Lymnaeidae were needed in the regression, although the permutation test suggested that its effect was nonsignificant (*p* = 0.074). The densities of Chironomidae and Lymnaeidae together explained 53.63% of the variation in the abundance of shorebirds at Wuliangsuhai Lake during the autumn migration (*r* ^2^ = 0.536, *p* = 0.046).

### 3.4. Environmental Factors Influencing the Abundance and Distribution of Benthic Macroinvertebrates

#### 3.4.1. Water Quality

A wide range of environmental gradients was evident across the three sampling sites, most noticeably salinity (*p* = 0.022, Table 2), ranging from brackish to supersaline (5,887–42,600 mg/L). A range of concentrations of Chl a (*p* = 0.024), TN (*p* = 0.014), and TP (*p* = 0.013) was also evident, with the concentrations at GSE (12.41, 4.36, and 0.282 mg/L for Chl a, TN, and TP, respectively) and XHZ (7.80, 12.4, and 0.356 mg/L for Chl a, TN, and TP, respectively) significantly higher than those at HK (4.04, 1.47, and 0.070 mg/L for Chl a, TN, and TP, respectively). The water temperature at GSE and XHZ was 3.00°C higher than at HK (*p* = 0.030). The range of dissolved oxygen concentrations was not significantly different among three sampling sites (76.8–96.92%, *p* = 0.377), nor was the pH (8.87–9.34, *p* = 0.187) (Table 2).

#### 3.4.2. Substrate Quality

The concentrations of N, P, and OM in the sediment varied significantly among the three sampling sites (*p* = 0.042, *p* < 0.001, and *p* < 0.001, respectively), with the concentration of N and OM highest at HK and the concentration of P highest at GSE. The concentrations of all the heavy metals (*p* < 0.001), except Hg (*p* = 0.051), were significantly different among the three sampling sites, with the concentrations at XHZ and GSE higher than those at HK. The concentrations of Li in the upper layer of the sediments were significantly higher than in the bottom layer (*p* = 0.010), whereas the concentrations of Co and Ni were higher in the deeper sediments (*p* = 0.036) (Table 3).

#### 3.4.3. Effects of Environmental Variables on Benthic Macroinvertebrates

**Figure 6:**
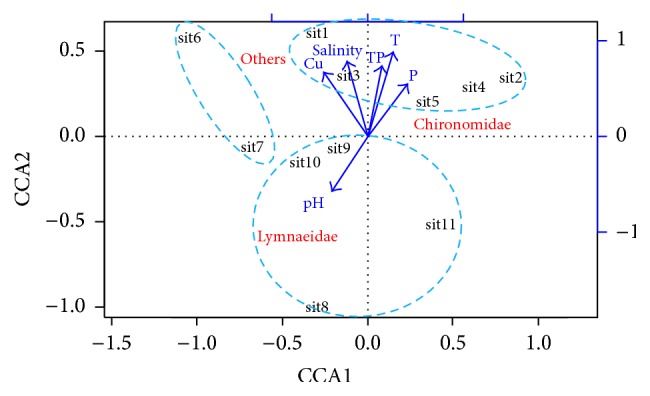
Plot of first two axes of canonical correspondence analysis (CCA) ordination based on the log-transformed benthic macroinvertebrate density data and environmental variables (arrows) at different sites.

Six environmental variables were included in the final CCA (Figure 6). The first two canonical axes collectively explained 87.5% of the variance in benthic macroinvertebrate distribution (60.2 and 27.3% for axes 1 and 2, respectively). Water temperature, salinity, TP, and Cu were significantly positively correlated with axis 2 (Table 4) and axis 2 was positively associated with Chironomidae (Figure 6). From Figure 6, it is clear that Chironomidae were mainly found at sampling sites with a higher water temperature and a higher concentration of both water TP and sediment P, but with a lower concentration of Cu and a lower water pH. By contrast, Lymnaeidae were largely found at sites with a higher water pH, but with a lower water temperature, lower water TP and salinity, and sediment P. Other macroinvertebrate species (e.g., the larvae of Tabanidae and Tubificidae) mainly occurred at sites with higher concentrations of Cu and higher salinity (Figure 6). The permutation test also indicated that there was a significant relationship between macroinvertebrate density and water/sediment quality (*p* = 0.013).

## 4. Discussion

### 4.1. Factors Influencing the Abundance and Distribution of Benthic Macroinvertebrates

The abundance of benthic macroinvertebrates was generally lower at sites that had poorer water and sediment quality, with the abundance decreasing from HK (near the outlet with higher water and sediment quality than other sites), to XHZ (near the drainage inlet with the highest concentrations of nutrients and pollutants). A similar tendency has been reported within the wetland, with the density and biomass of macroinvertebrates increasing with increasing distance from the main sources of pollution [33]. Among the recorded benthic macroinvertebrates, Chironomidae and Lymnaeidae had higher abundances at sites close to the outlet of the wetland where the salinity, TN, TP, and heavy metal (in particular Cu) concentrations were much lower than at XHZ and GSE. The pattern was particularly clear for Lymnaeidae, which was absent from the majority of sampling plots at XHZ. This indicates that Lymnaeidae have a relatively higher requirement for water and substrate quality than Chironomidae and that high levels of salinity and TP or heavy metals could restrict the survival of Lymnaeidae [34]. Some species of Chironomidae larvae can tolerate very high levels of pollution [35, 36] and they often become the dominant group of benthic invertebrates in polluted water bodies such as Wuliangsuhai Lake.

Benthic invertebrates are strongly affected by environmental stress, such as inorganic contaminants [37]—for example, the accumulation of heavy metals in sediments could lead to the death of benthic invertebrates [38]. Several effects of metal contamination on benthic communities have been documented, including decreased density [39, 40], a reduction in the number of sensitive taxa [41], and changes in the distribution patterns of species [42]. Our study suggested that water temperature, salinity, TP, and Cu concentrations in sediments were the most important variables in determining the density and distribution of benthic macroinvertebrate. Other variables, such as water pH and the P concentration in the sediment, might be less important for benthic macroinvertebrate at Wuliangsuhai Lake. pH does not play an important part in determining the distribution of Lymnaeidae in natural water bodies [34]. Although the CCA indicated that the sediment P concentration did not have a positive relationship with the density of benthic macroinvertebrates, this result should be interpreted with caution. Because the sediment P and water TP were highly positively correlated, which is consistent with the study of Sun et al. [43], the negative relationship between the water column TP and the abundance of benthic macroinvertebrates might imply a negative relationship between the sediment P and the abundance of benthic macroinvertebrates. Previous studies have shown that the amount of sediment P at Wuliangsuhai Lake may be a threat to the benthic communities and may have exacerbated the eutrophic level of the water body [44].

The concentration of Cu had a more significant effect on the benthic macroinvertebrates than other heavy metals. In a study of benthic fauna and pollutant levels in Norwegian fjords, Brage [45] also found that Cu had the highest deleterious effect. The high Cu level in the top 5 cm of sediments sampled from GSE and XHZ was evidence of slight to moderate heavy metal pollution [46]. The substrate type also played an important part in determining the species, abundance, and distribution of benthic invertebrates [47]. Chironomidae larvae preferred muddy substrates, whereas Lymnaeidae preferred sandy substrates at Wuliangsuhai Lake.

The loading of nutrients (especially TP) and heavy metals (in particular Cu) into the aquatic environment has increased with the intensity of human activities. The input of nutrients into Wuliangsuhai Lake is mainly from irrigation drainage water [20] and the source of heavy metals is mainly from industrial wastewater and domestic sewage around the wetland [48]. As a result, the diversity and distribution of benthic macroinvertebrates may have changed. Some species may even disappear locally when pollution exceeds their tolerance level. For example, Lymnaeidae were absent from most sampling plots at XHZ. As demonstrated in this and many previous studies [49–51], shorebirds positively follow the distribution pattern of benthic macroinvertebrates and therefore changes in the benthic macroinvertebrate communities might eventually lead to the functional loss of Wuliangsuhai Lake as a stopover site for migratory shorebirds. Reducing the nutrient loadings and controlling water pollution are crucial for waterbird conservation at Wuliangsuhai Lake.

### 4.2. Distribution of Shorebirds and Relationship with Benthic Macroinvertebrates

Our results showed that the distribution of shorebirds at Wuliangsuhai Lake was not random, with the greatest abundance of shorebirds at GSE, the main foraging habitat during peak migration. However, in mid-October 2011, after the peak season when most shorebirds had left, HK provided the main foraging habitat for late migrants [12] (Figure 2). These two main foraging areas also had the highest abundance of benthic macroinvertebrates (Figure 3). Numerous studies have shown positive relationships between shorebirds and prey abundance or biomass, but the strength of the relationship varies among studies [52, 53]. Our study confirmed the numerical relationship between shorebirds and benthic macroinvertebrates within Wuliangsuhai Lake, even though the relationship was not very strong (the *r* ^2^ value of the multiple linear regression was moderate 53.63%), which may be due to the relatively small sample size (only during autumn migration). Prey density alone is unlikely to account for all the variation in bird density [54]—for example, GSE had the highest average shorebird density, but not the highest average prey density. Other variables, such as vegetation cover [55] and prey availability, which are associated with water depth [56], should be incorporated into future studies. Determination of the dynamic interaction between shorebirds and the density of benthic macroinvertebrates (e.g., sites with more abundant macroinvertebrates would attract more shorebirds, which, in turn, could suppress the abundance of macroinvertebrates) would give a better insight into the ecological communities and help in the conservation of the wetland ecosystem.

Many shorebird species are long-distance migrants and require high-quality stopover sites to rest and refuel for their next journey. One of the most important factors is the availability of food at the stopover sites [57]. It is also known that the interactions among shorebirds, their prey, and the environment are important [6, 58–61] and provide basic information for the management of shorebird habitats. The populations of long-distance migratory shorebirds around the world are decreasing [62] and some of the steepest and most widespread declines have been observed in the East Asian-Australasian Flyway [63]. Most of the earlier studies have focused only on shorebirds and their habitats in the coastal zones of the flyway [5, 64, 65] and there is little information available on inland wetlands. Our study focused on the numerical relationship between shorebirds and benthic macroinvertebrates in a temperate arid wetland and provides important data for future studies.

## 5. Conclusions

The abundance of both shorebirds and benthic macroinvertebrates at Wuliangsuhai Lake varied significantly in space and time. Environmental factors, including the water and substrate quality, had strong impacts on the abundance and distribution of benthic macroinvertebrates. The shorebirds positively followed the distribution patterns of the benthic macroinvertebrates. Thus, the numerical relationship between shorebirds and benthic macroinvertebrates within Wuliangsuhai Lake was confirmed. Our findings suggest that reducing the nutrient and heavy metal loadings is crucial in maintaining the ecological function of Wuliangsuhai Lake as a foraging and stopover habitat for migratory shorebirds.

## Figures and Tables

**Table 1:** Mean ± SD benthic macroinvertebrate densities from the counts at Wuliangsuhai Lake during the autumn migration in 2011 (*n* = 33).

Time period	Chironomidae	Lymnaeidae	Others
Peak migration	5,168 ± 13,392	205 ± 490	90 ± 182
Postpeak migration	1,303 ± 1,842	0 ± 0	0 ± 0

**Table 2:** Mean ± SD values of the principal water quality characteristics across the three sampling sites within the lake: Gesuer (GSE), Hekou (HK), and Xiaohaizi (XHZ) (*n* = 11).

Sampling site	Water temperature (°C)	pH	Dissolved oxygen (%)	Chlorophyll a (mg/L)	Salinity (mg/L)	Total nitrogen (mg/L)	Total phosphorus (mg/L)
GSE	19.32 ± 0.96	8.87 ± 0.10	96.92 ± 15.34	12.41 ± 2.30	29,660 ± 13,076	4.36 ± 0.59	0.282 ± 0.032
HK	15.98 ± 1.15	9.34 ± 0.54	76.8 ± 15.0	4.04 ± 1.04	5,887 ± 537	1.47 ± 0.09	0.070 ± 0.011
XHZ	19.15 ± 0.75	9.04 ± 0.02	83.3 ± 16.7	7.80 ± 3.02	42,600 ± 2,500	12.4 ± 1.0	0.356 ± 0.000

**Table 3:** Mean ± SD values of the principal substrate quality characteristics across the three sampling sites within the lake (Gesuer (GSE), Hekou (HK), and Xiaohaizi (XHZ)) in sediments from depths of 0–5 and 5–10 cm (*n* = 15, 12, and 6 for GSE, HK, and XHZ, respectively).

Substrate characteristic	GSE		HK		XHZ	
	0–5 cm	5–10 cm	0–5 cm	5–10 cm	0–5 cm	5–10 cm
Organic matter (%)	1.33 ± 0.62	1.17 ± 0.43	3.22 ± 1.36	1.97 ± 0.85	2.30 ± 1.03	1.59 ± 0.54
N (mg/kg)	1362 ± 703	717 ± 202	1508 ± 637	1403 ± 863	1134 ± 370	1001 ± 242
P (mg/kg)	916.2 ± 69.6	972.30 ± 83.84	835.85 ± 59.16	818.98 ± 54.06	804.53 ± 29.72	866.23 ± 54.65
As (mg/kg)	21.14 ± 5.81	25.98 ± 7.56	9.20 ± 1.99	12.30 ± 3.60	29.68 ± 4.26	35.30 ± 5.98
Co (mg/kg)	10.67 ± 2.30	12.20 ± 2.44	8.92 ± 1.39	9.46 ± 1.13	11.49 ± 0.52	12.81 ± 0.59
Cu (mg/kg)	26.63 ± 6.19	30.93 ± 10.82	19.69 ± 4.10	21.12 ± 3.51	33.26 ± 3.84	37.20 ± 11.71
Li (mg/kg)	40.60 ± 5.47	44.46 ± 6.72	37.08 ± 8.14	37.44 ± 6.51	49.78 ± 2.20	51.97 ± 3.27
Ni (mg/kg)	30.28 ± 6.72	34.61 ± 7.59	26.74 ± 4.64	27.94 ± 4.29	31.25 ± 1.48	34.63 ± 2.89
Hg (mg/kg)	0.01 ± 0.00	0.02 ± 0.00	0.01 ± 0.01	0.01 ± 0.00	0.01 ± 0.00	0.01 ± 0.00

**Table 4:** Correlation coefficients of environmental variables with the first two axes of canonical correspondence analysis.

Environmental variable	Axis	
	1	2
pH	−0.58418	−0.81162
Water temperature	0.31746	0.94827^*∗∗*^
Salinity	−0.29459	0.95562^*∗*^
Total phosphorus	0.20732	0.97827^*∗*^
P	0.63746	0.77048
Cu	−0.60334	0.79748^*∗*^

Significance at ^*∗*^ *p* < 0.05 and ^*∗∗*^ *p* < 0.01.
